# Fructose-Rich Diet Affects Mitochondrial DNA Damage and Repair in Rats

**DOI:** 10.3390/nu9040323

**Published:** 2017-03-24

**Authors:** Federica Cioffi, Rosalba Senese, Pasquale Lasala, Angela Ziello, Arianna Mazzoli, Raffaella Crescenzo, Giovanna Liverini, Antonia Lanni, Fernando Goglia, Susanna Iossa

**Affiliations:** 1 Department of Science and Technology, University of Sannio, 82100 Benevento, Italy; federica.cioffi@unisannio.it (F.C.); pasquale.lasala@gmail.com (P.L.); 2 Department of Environmental, Biological and Pharmaceutical Sciences and Technologies, University of Naples II, 81100 Caserta, Italy; rosalba.senese@unina2.it (R.S.); angela.ziello@gmail.com (A.Z.); antonia.lanni@unina2.it (A.L.); 3 Department of Biology, University of Naples “Federico II”, 80100 Napoli, Italy; arimazzoli@hotmail.it (A.M.); rcrescen@unina.it (R.C.); liverini@unina.it (G.L.); susiossa@unina.it (S.I.)

**Keywords:** fructose-rich diet, mitochondrial biogenesis, mitochondrial DNA (mtDNA), oxidative damage, repair mechanisms

## Abstract

Evidence indicates that many forms of fructose-induced metabolic disturbance are associated with oxidative stress and mitochondrial dysfunction. Mitochondria are prominent targets of oxidative damage; however, it is not clear whether mitochondrial DNA (mtDNA) damage and/or its lack of repair are events involved in metabolic disease resulting from a fructose-rich diet. In the present study, we evaluated the degree of oxidative damage to liver mtDNA and its repair, in addition to the state of oxidative stress and antioxidant defense in the liver of rats fed a high-fructose diet. We used male rats feeding on a high-fructose or control diet for eight weeks. Our results showed an increase in mtDNA damage in the liver of rats fed a high-fructose diet and this damage, as evaluated by the expression of DNA polymerase γ, was not repaired; in addition, the mtDNA copy number was found to be significantly reduced. A reduction in the mtDNA copy number is indicative of impaired mitochondrial biogenesis, as is the finding of a reduction in the expression of genes involved in mitochondrial biogenesis. In conclusion, a fructose-rich diet leads to mitochondrial and mtDNA damage, which consequently may have a role in liver dysfunction and metabolic diseases.

## 1. Introduction

Over the last few decades, the daily intake of fructose, either free or as high-fructose corn syrup, has markedly increased [1]. These forms of fructose are used in the food industry for their enhanced sweetness, palatability, solubility, lower cost, and high production efficiency compared to sugar [2]. However, the increase in fructose consumption has coincided with a rise in the incidence of obesity, metabolic syndrome, and type 2 diabetes [3,4]. Several researchers have even suggested that increased fructose consumption has directly contributed to the obesity and type 2 diabetes epidemic [3,4].

Previous studies have shown that fructose-rich diets can induce many features of metabolic syndrome, including hypertension, insulin resistance, abdominal obesity, hepatic steatosis, endothelial dysfunction, and inflammation [5,6,7,8,9,10]. Fructose is a highly lipogenic substrate which can induce profound metabolic alterations in the liver [11], where 90% of ingested sugar is metabolized [12]. Although the mechanisms underlying fructose-mediated metabolic disease are not entirely understood, previous studies have suggested a causative role for oxidative stress [13,14,15,16], an imbalance between reactive oxygen species (ROS) generation and removal by antioxidant defense systems. Mitochondria produce ROS through the respiratory chain, but are also equipped with antioxidant enzymes, thereby participating in redox regulation. Since oxidative stress is considered a key factor in the development of metabolic alterations [17,18], the marked effect of fructose on systemic oxidative stress could explain its role in the pathophysiology of insulin resistance and metabolic syndrome [19,20,21]. In agreement with this, in rats fed a high-fructose diet, we have previously found hepatic insulin resistance together with hepatic mitochondrial oxidative damage, both in the lipid and in the protein component, as well as decreased activity of antioxidant defense [11]. In addition, Mamikutty et al. [22] found that after eight weeks of high fructose consumption, rats developed several features of metabolic syndrome, together with mitochondrial structural alterations. Therefore, it seems that there is a strong association between the effect of fructose at the cellular level and the mitochondrial compartment. However, information on the possible mechanism by which mitochondrial function is altered by fructose feeding is lacking. Mitochondria contain a double-stranded, circular DNA that encodes many proteins essential for ATP production. Malfunction of the antioxidant defense system leads to oxidative attack, resulting in mtDNA damage, which in turn can lead to a decline in mitochondrial functions and turnover, and in an impairment of appropriate stress responses that monitor and maintain their quality. Taking into account the above considerations, we believe that it becomes relevant to investigate the consequences that a fructose-rich diet may have on oxidative stress and on mtDNA damage in a metabolically very important organ such as the liver.

Therefore, the purpose of this study was to evaluate in the liver of fructose-fed rats: (i) a possible induction of mtDNA damage; (ii) changes in the expression of a specific enzyme associated with the mtDNA repair mechanism; and (iii) a possible involvement of mechanisms underlying mitochondrial biogenesis and the cellular antioxidant defense system.

## 2. Materials and Methods

### 2.1. Animals and Treatments

Male Sprague-Dawley rats (Charles River, Calco (LC), Italy) of about 100 days of age were caged singly in a temperature-controlled room (23 ± 1 °C) with a 12 h light/dark cycle (6:30 a.m.–6:30 p.m.). Animal treatment, housing, and euthanasia met the guidelines set by the Italian Health Ministry. All experimental procedures were also approved by “Comitato Etico-Scientifico per la Sperimentazione Animale” of the University “Federico II” of Naples, Italy.

Rats were divided in two groups, each with the same mean body weight (470 ± 10 g), and either fed a control or a fructose-rich diet (composition of the two diets is shown in Table 1), known to induce early signs of obesity within eight weeks of treatment [7,8,11]. Briefly, rats were pair-fed for eight weeks, by giving them the same amount of diet, both as weight and as caloric content and each rat consumed the full portion of the diet. During the treatment, body weight, food, and water intake were monitored daily. At the end of the experimental period, the rats were euthanized by decapitation, and blood and liver samples were collected.

### 2.2. Plasma Parameters

Plasma concentrations of alanine aminotransferase (ALT), and aspartate aminotransferase (AST) were measured by colorimetric enzymatic method using commercial kits (SGM Italia, Rome, Italy).

On plasma samples, 8-hydroxy-2′-deoxyguanosine (8-OHdG), a critical biomarker of oxidative stress [23] was quantified using a DNA/RNA Oxidative Damage ELISA kit (Cayman Chemical Company, Ann Arbor, MI, USA) according to the manufacturer’s protocol. Plasma samples were analyzed in duplicate. Standard 8-OHdG was assayed over a concentration range of 10.3–3000 pg·mL^−1^ in duplicate for each experiment.

### 2.3. Genomic DNA Isolation

Total liver DNA was extracted using the Genomic-tip 20/G kit (Qiagen, Valencia, CA, USA) according to the manufacturer’s protocol. The quantification of the purified genomic DNA and PCR products was performed fluorometrically using the Picogreen ds DNA reagent (Invitrogen, Milan, Italy).

### 2.4. Quantitative Polymerase Chain Reaction (QPCR)

QPCR was performed on liver DNA extracts as previously described [24] with the following modification: the PCR amplification was done using the Ranger DNA Polymerase with the appropriate premixes (Bioline Ltd., London, UK). Two pairs of PCR primers were employed:

mtDNA long fragment (13.4 Kbp): 5′-AAAATCCCCGCAAACAATGACCACCC-3′ (sense)/5′-GGCAATTAAGAGTGGGATGGAGCCAA-3′ (anti-sense);
mtDNA short fragment (235 bp): 5′-CCTCCCATTCATTATCGCCGCCCTGC-3′ (sense)/5′-GTCTGGGTCTCCTAGTAGGTCTGGGAA-3′ (anti-sense).

For amplification of the mtDNA long fragment, the standard thermocycler program included initial denaturation at 94 °C for 1 min, 18 cycles of 94 °C for 15 s, 65 °C for 12 min, and final extension at 72 °C for 10 min. To amplify the short mtDNA fragment (235 bp), the same program was used except the extension temperature was changed to 60 °C. DNA damage was quantified by comparing the relative efficiency of amplification of the long mtDNA fragment normalized to the amplification of the small mtDNA fragment. QPCR products were quantified using PicoGreen dye and a fluorescence plate reader in the same manner as the template DNA. The resulting values were converted to relative lesion frequencies per 10 Kbp DNA by applying the Poisson distribution.

### 2.5. mtDNA Copy Number

Relative mtDNA copy numbers were measured liver genomic DNA by real-time quantitative PCR (qRT-PCR) and corrected by simultaneous measurement of nuclear DNA. We examined the amplification of mitochondrial cytochrome c oxidase subunit II (COII, mitochondrial-encoded gene) and β-actin (nuclear-encoded gene). The primer sequences used were as follows:

COII: 5′-TGAGCCATCCCTTCACTAGG-3′ (sense)/5′-TGAGCCGCAAATTTCAGAG-3′(anti-sense);
β-actin: 5′-CTGCTCTTTCCCAGATGAGG-3′ (sense)/5′-CCACAGCACTGTAGGGGTTT-3′ (anti-sense).

The threshold cycle (Ct) reflects the cycle number at which a fluorescence signal within a reaction crosses a threshold. In our study, the average Ct values of nuclear DNA and mtDNA were obtained for each case. mtDNA content was calculated using ΔCt = average Ct_nuclear DNA_ − average Ct_mtDNA_ and then was obtained using the formula mtDNA content = 2 (2ΔCt).

### 2.6. mRNA Expression

Total liver RNA was isolated using TRIzol^®^ reagent (Invitrogen) according to the manufacturer′s protocol. Tissue/TRIzol^®^ mixtures were homogenized using an Ultra Turrax homogenizer while keeping the viscosity of the solution to a minimum to ensure effective inactivation of endogenous RNAse activity. RNA samples were subjected to DNAse treatment to remove genomic DNA contamination. A total of 1 μg of total RNA was used to generate cDNA in a 20-μL reaction volume using Superscript II Reverse Transcriptase (HT Biotechnology, Cambridge, UK). PCR primers were designed using Primer Express version 2.0 (Invitrogen). We examined the mRNA expression of DNA polymerase γ (Polg), Peroxisome proliferator-activated receptor γ coactivator-1α (Pgc1α), Nuclear respiratory factor 1 (Nrf1) and Mitochondrial transcription factor A (Tfam). β-actin mRNA expression was used for normalization. Primers used were as follows:

β-actin: 5′-CTGCTCTTTCCCAGATGAGG-3′ (sense)/5′-CCACAGCACTGTAGGGGTTT-3′ (anti-sense);
Polg: 5′-GAAGAGCGTTACTCTTGGACCAG-3′ (sense)/5′-AACATTGTGCCCCACCACTAAC-3′ (anti-sense);
Pgc1α: 5′-GTCAACAGCAAAAGCCACAA-3′ (sense)/5′-GTGTGAGGAGGGTCATCGTT-3′ (anti-sense);
Nrf1: 5′-CTGATGGCCATTACATGTGG-3′ (sense)/5′-GTAAAGCCCGGAAGGTTCTT-3′ (anti-sense);
Tfam: 5′-CAACAGGGAAGAAACGGAAA-3′ (sense)/5′-GTGGCTCTGAGTTTCCGAAG-3′ (anti-sense).

An equivalent of 25 ng of total RNA was subsequently used in the amplification with 50 nmol of gene-specific primers and 4 mL of iTaq Universal SYBR Green mix (Bio-Rad Laboratories, Hercules, CA, USA) in a total volume of 8 μL using standard cycle parameters on a Bio-Rad iQ5.

### 2.7. Preparation of Hepatic Homogenate and Isolated Mitochondria

Rat liver was gently homogenized in 10 volumes of isolation medium consisting of 220 mmol mannitol, 70 mmol sucrose, 20 mmol Tris-HCl, and 1 mmol EDTA at pH 7.4 (Sigma-Aldrich, St. Louis, MO, USA). Aliquots of homogenate were withdrawn for further measurements while the remaining homogenate was centrifuged at 500× *g* for 10 min at 4 °C and the resulting supernatant was centrifuged at 3000× *g* 10 min at 4 °C. The mitochondrial pellet was then washed twice and solubilized in a minimal volume of RIPA buffer (50 mmol Tris-HCl (pH = 7.4), 150 mmol NaCl, 1% NP-40, 0.1% SDS, 2 mmol EDTA, 0.5% sodium deoxycholate) until addition of protease/phosphatase inhibitors and kept on ice. The mitochondrial protein concentration was determined using the Bio-Rad DC method and the mitochondrial samples were then used for Western blot analysis of catalase content.

### 2.8. Hepatic Lipid Peroxidation

Lipid peroxidation was determined according to Fernandes et al. [25] in liver homogenates prepared as described above, by measuring thiobarbituric acid reactive substances (TBARS), using the thiobarbituric acid assay. Aliquots of hepatic homogenates were added to 0.5 mL of ice-cold 40% trichloroacetic acid. Then, 2 mL of 0.67% of aqueous thiobarbituric acid containing 0.01% of 2,6-di-tert-butyl-p-cresol was added. The mixtures were heated at 90 °C for 15 min, then cooled in ice for 10 min, and centrifuged at 850× *g* for 10 min. The supernatant fractions were collected and lipid peroxidation was estimated spectrophotometrically at 530 nm. The amount of TBARS formed was calculated using a molar extinction coefficient of 1.56 × 10^5^ mol^−1^·cm^−1^ and expressed as nmol TBARS·g^−1^ tissue.

### 2.9. Hepatic Myeloperoxidase (MPO) Activity

MPO activity was assessed in liver samples as reported by Kim et al. [26]. Briefly, tissue samples (100 mg) were homogenized in 1 mL of hexadecyltrimethylammoniumbromide (HTAB) buffer (0.5% HTAB in 50 mmol phosphate buffer, pH 6.0) and centrifuged at 13,400× *g* for 6 min at 4 °C. Then, 10 μL of supernatant were combined with 200 μL of 50 mmol phosphate buffer, pH 6.0, containing 0.167 mg·mL^−1^ 0-dianisidine hydrochloride and 1.25% hydrogen peroxide. The change in absorbance at 450 nm was measured and one unit of MPO activity was defined as that degrading 1 μmol of peroxide per minute at 25 °C.

### 2.10. Western Blotting

Liver tissue was homogenized in lysis buffer containing 20 mmol Tris-HCl (pH 7.5), 150 mmol NaCl, 1 mmol EDTA, 1 mmol EGTA, 2.5 mmol Na_2_H_2_P_2_O_7_, 1 mmol b-CH_3_H_7_O_6_PNa_2_, 1 mmol Na_3_VO_4_, 1 mmol PMSF 1 mg·mL^−1^ leupeptin, and 1% Triton X-100 (Sigma-Aldrich, St. Louis, MO, USA) using an Ultra Turrax homogenizer and then centrifuged at 15,000× *g* in a Beckman Optima TLX Ultracentrifuge (Beckman Coulter S.P.A., Milan, Italy) for 15 min at 4 °C. The supernatants were then ultracentrifuged at 40,000× *g* in a Beckman Optima TLX ultracentrifuge for 15 min at 4 °C. The protein concentration in supernatants and cleared lysates was determined using the Bio-Rad DC method. The protein levels of POLG, PGC1α, NRF1 and TFAM were determined in the supernatants of ultracentrifuged lysates using polyclonal antibodies (Novus Biologicals, Littleton, CO, USA; Millipore, Billerica, MA, USA; Abcam, Cruz Biotechnology, Santa Cruz, CA, USA, respectively). β-actin antibody (Sigma-Aldrich) was used as control.

Catalase protein levels were measured on protein extracts from isolated liver mitochondria using a polyclonal antibody (Sigma-Aldrich) and a voltage-dependent anion channel (Santa Cruz Biotechnology, Santa Cruz, CA, USA) was used as control.

### 2.11. Statistical Analysis

Results are expressed as means ± SEM. Statistical analyses were performed using a two-tailed, unpaired Student’s *t*-test. Differences were considered statistically significant at *p* < 0.05.

## 3. Results

### 3.1. Effect of Fructose-Rich Diet on Hepatic Functionality

As shown in Table 2, in fructose-fed rats, plasma levels of ALT and AST, biochemical indicators of hepatic damage, and hepatic levels of TBARS, markers of lipid peroxidation, were significantly higher compared to the controls. In addition, fructose-fed rats exhibited a significant increase in hepatic MPO activity compared to the controls. The determination of MPO activity can be used as a surrogate marker of inflammation, since it has been shown that the activity of MPO solubilized from the inflamed tissue is directly proportional to the number of neutrophils seen in histologic sections [27].

### 3.2. Effect of Fructose-Rich Diet on Plasma 8-OHdG Concentration

**Figure 1:**
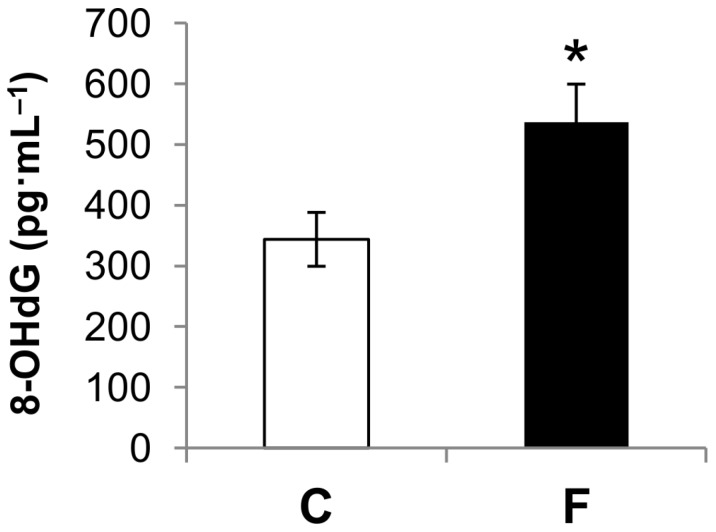
Effect of fructose-rich diet on plasma 8-hydroxy-2′-deoxyguanosine (8-OHdG) levels. Values represent means ± SEM from five rats in each group. C: control diet; F: fructose-rich diet. * *p* < 0.05 versus C rats.

As shown in Figure 1, administration of a fructose-rich diet for eight weeks resulted in significantly increased levels of plasma 8-OHdG (+56%) compared to control rats. The reaction of intracellular ROS with DNA results in numerous forms of base damage, and 8-OHdG is one of the most abundant and most studied lesions generated. So, 8-OHdG has been used as an indicator of oxidative DNA damage in vivo and in vitro [23,28]. The above result of increased levels of plasma 8-OHdG in rats fed a fructose-rich diet suggests that such treatment induces an increase in DNA damage.

### 3.3. Effect of Fructose-Rich Diet on Mitochondrial Catalase Expression

**Figure 2:**
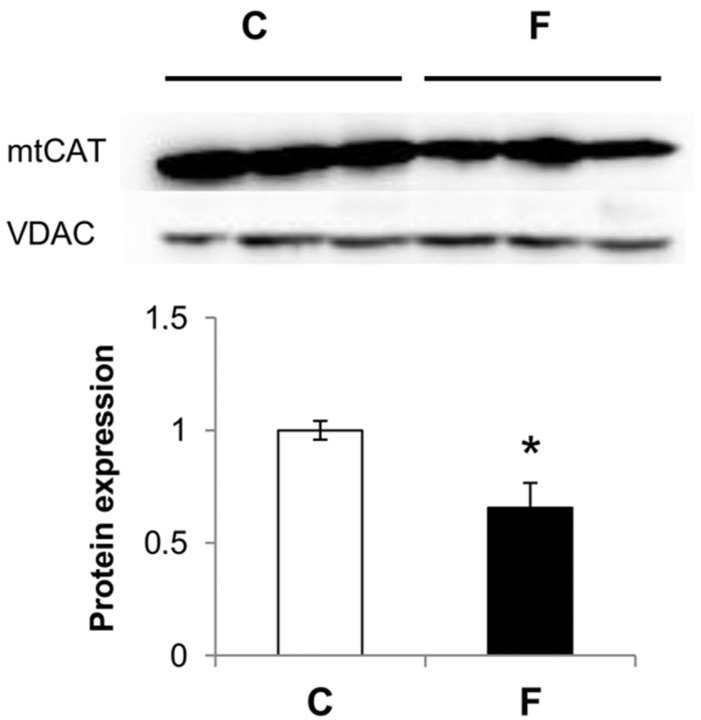
Effect of fructose-rich diet on catalase expression in isolated liver mitochondria. Upper panel: representative Western blot with mitochondrial catalase (mtCAT) antibody using voltage-dependent anion channel (VDAC) as an internal control. Lower panel: quantitative analysis of Western blot. Values are presented as means ± SEM from six rats in each group. C: control diet; F: fructose-rich diet. * *p* < 0.05 versus C rats.

As shown in Figure 2, catalase protein levels in rat liver mitochondria were decreased by 35% in rats fed a fructose-rich diet compared to controls.

### 3.4. Effect of Fructose-Rich Diet on mtDNA Damage and Copy Number

**Figure 3:**
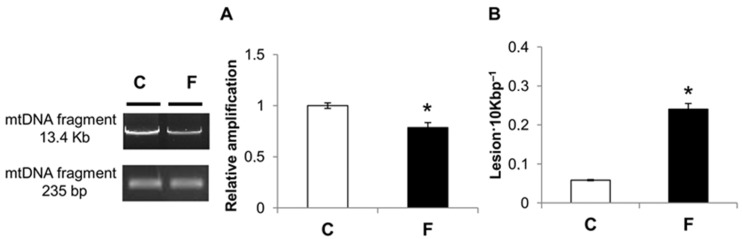
Effect of fructose-rich diet on mtDNA damage and lesion frequency. (**A**) mtDNA damage was evaluated in the liver by amplifying long (13.4 Kbp) and short (235 bp) mtDNA fragments by QPCR; (**B**) Frequency of mtDNA lesions per 10 Kbp per strand. Values are presented as means ± SEM from four rats in each group. C: control diet; F: fructose-rich diet. * *p* < 0.05 versus C rats.

QPCR was used to measure the levels of hepatic mtDNA oxidative damage. In rats fed a fructose-rich diet, the relative amplification of long (13.4 Kbp) mtDNA fragments was significantly reduced by 22% compared to control rats (Figure 3a). Liver mtDNA from rats fed a fructose-rich diet contained significantly more mtDNA lesions (0.24 lesion·10 Kbp^−1^) compared to control rats (0.058 lesion·10 Kbp^−1^) (Figure 3b).

**Figure 4:**
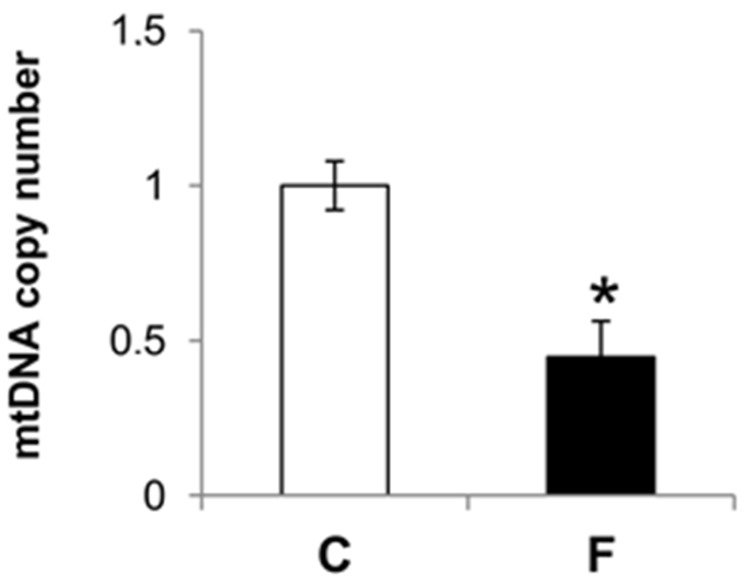
Effect of a fructose-rich diet on mtDNA copy number. mtDNA copy number was assessed by quantitative reverse transcription polymerase chain reaction in 10 ng of genomic liver DNA using primers for mtCOII. Expression was normalized using nuclear β-actin as an internal control. Values are presented as means ± SEM from four rats in each group. C: control diet; F: fructose-rich diet. * *p* < 0.05 versus C rats.

These results demonstrate that in rats fed a fructose-rich diet for eight weeks, there was a significant increase in oxidative damage to the mtDNA. Moreover, the mtDNA copy number was significantly reduced with the fructose-rich (−56%) versus control diet (Figure 4).

### 3.5. Effect of Fructose-Rich Diet on Mitochondrial POLG

**Figure 5:**
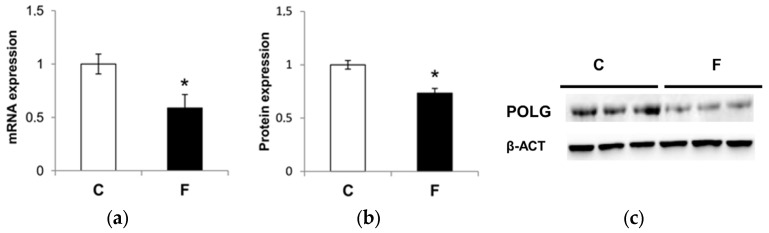
Effect of fructose-rich diet on DNA polymerase γ (POLG) expression. (**a**) POLG mRNA expression was measured by quantitative reverse transcription polymerase chain reaction using β-actin as an internal control. Values represent means ± SEM from four rats in each group; (**b**,**c**) Quantitative analysis and representative Western blot with POLG antibody using β-actin as an internal control. Values represent means ± SEM from nine rats in each group. C: control diet; F: fructose-rich diet. * *p* < 0.05 versus C rats.

POLG is an important enzyme involved in mtDNA repair and replication. To investigate whether a fructose-rich diet caused changes in the POLG expression, qRT-PCR and Western blot analysis were performed. In rats fed a fructose-rich diet, the POLG mRNA expression (Figure 5a) and protein levels (Figure 5b) were found to be significantly decreased by 42% and 27%, respectively, compared to control rats.

### 3.6. Effect of Fructose-Rich Diet on Mitochondrial Biogenesis

Hepatic mitochondrial biogenesis was evaluated by measuring the mRNA and protein levels of PGC1α, NRF1, and TFAM. PGC1α is one of the most important coactivators of mitochondrial biogenesis, which controls many aspect of oxidative metabolism, through co-activation and enhancement of the expression and activity of several transcription factors, including NRF1 [29,30]. PGC-1alpha is also indirectly involved in regulating the expression of mtDNA transcription via the increased expression of TFAM, which is coactivated by NRF1 [29,31].

**Figure 6:**
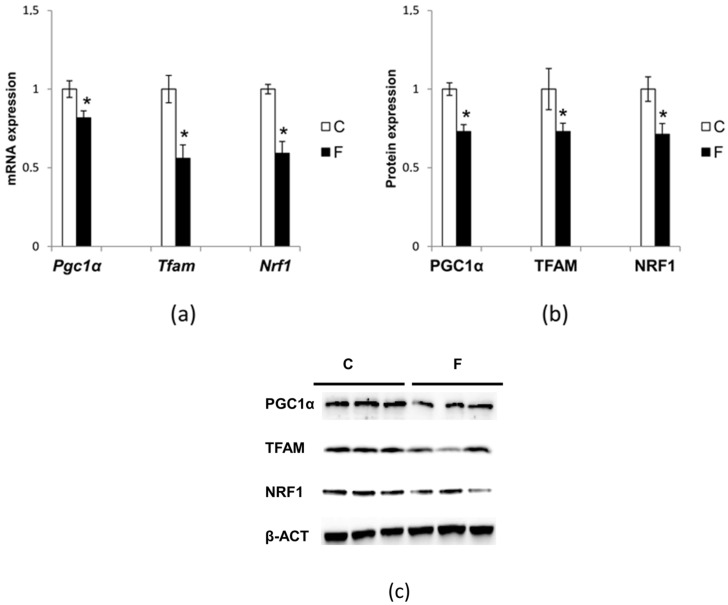
Effect of fructose-rich diet on peroxisome proliferator-activated receptor γ coactivator-1α (PGC1α), nuclear respiratory factors (NRF)-1, and mitochondrial transcription factor A (TFAM) expression. (**a**) Pgc1α, Nrf1 and Tfam mRNA expression was measured by quantitative reverse transcription polymerase chain reaction using β-actin as an internal control. Values are presented as means ± SEM from four rats in each group. C: control diet; F: fructose-rich diet. * *p* < 0.05 versus C rats; (**b**,**c**) quantitative analysis and representative Western blot with PGC1α, NRF1 and TFAM antibodies using β-ACTIN as an internal control. Values are presented as means ± SEM from six rats in each group. C: control diet; F: fructose-rich diet. * *p* < 0.05 versus C rats.

In rats fed fructose-rich diets, Pgc1α, Nrf1, and Tfam (Figure 6a) mRNA expression was significantly reduced by 19%, 41% and 43%, respectively, compared to control rats. Regarding the protein levels (Figure 6b), the reductions were 27%, 29% and 27%, respectively.

## 4. Discussion

In the present paper we show that long-term fructose intake is associated with increased systemic oxidative stress, as well as with marked oxidative alterations in liver cells, and in particular in hepatic mitochondria. 

Further, 8-OHdG is an abundant base modification in mammalian DNA, the levels of which increase with oxidative stress [28], and it is therefore widely recognized as a biomarker of the in vivo total systemic oxidative stress [23]. Herein, we found that 8-OHdG levels were markedly increased in rats fed a fructose-rich diet (Figure 1), thus indicating the condition of increased oxidative stress at the whole-body level induced by the high intake of fructose.

In this work, we found that chronic intake of fructose is also associated with various signs of liver damage, such as increased lipid peroxidation, inflammation and cellular necrosis. Fructose intake negatively impacts liver function, since absorbed monosaccharides are firstly sent to the liver via portal blood, and up to 90% of ingested fructose is metabolized in the liver [12]. Therefore, it is conceivable that the massive flow of fructose and its handling in the liver causes a metabolic injury to the tissue.

There is accumulating evidence that fructose promotes ROS imbalance via the simultaneous enhancement of ROS production and the down-regulation of the antioxidant defense mechanisms, and the consequence is widespread damage to biological macromolecules, namely lipid peroxidation, protein oxidation, and DNA base modification. Our previous studies have demonstrated that in rats fed a fructose-rich diet for eight weeks, hepatic mitochondria showed signs of oxidative damage, both in the lipid and in the protein component, together with the decreased activity of superoxide dismutase (SOD), one of the enzymatic components of the antioxidant defense system of mitochondria [11]. Another important member of the antioxidant system that protects mitochondria from ROS damage is mitochondrial catalase. Previous studies have identified the presence of catalase in mitochondria and its key role in oxidant defense [32,33,34]. In particular, these studies showed that mitochondrial catalase provides better protection than cytosolic catalase against H_2_O_2_-induced injury, oxidative damage, and mtDNA deletion [32,33,34]. Our present data of the decreased expression of catalase in rats fed a high-fructose diet (Figure 2) further support previous reports of increased oxidative stress in the hepatic mitochondrial compartment of rats fed fructose-rich diets [11]. In addition, it seems that there is a general decrease in the hepatic mitochondrial antioxidant systems induced by fructose, at variance with results obtained on fructose-fed fruit flies, where it has been shown that fructose feeding alters the activities of SOD and catalase in opposite ways [35]. Investigations on the antioxidant systems of the other cellular compartments could be useful to obtain a more complete picture. 

It is well known that mtDNA is very sensitive to oxidative damage because it is located close to the inner mitochondrial membrane, where ROS are generated, and also because of the absence of protective histones and fewer repair mechanisms. Despite this, data on the effect of a diet rich in fructose on mtDNA are lacking. Since the liver is the main tissue involved in fructose processing, we evaluated the degree of oxidative damage to liver mtDNA, and the mtDNA repair mechanisms. Our results showed an increase in oxidative mtDNA lesions in the liver of rats fed a fructose-rich diet, coupled with a decrease in the mitochondrial repairing capacity, as evaluated by POLG expression, (Figure 4 and Figure 5). Damage to mtDNA could lead to mutations during replication, possibly resulting in further important implications for cell physiology.

QPCR used in the present study was based on the principle that lesions present in the DNA block the progression of thermostable DNA polymerase on the template, resulting in decreased DNA amplification relative to undamaged DNA (amplification inversely proportional to the amount of damage). To exclude the possibility that the reduction in 13.4 Kbp mtDNA fragment amplification resulted from the loss of mtDNA molecules, we also amplified a small 235 bp mtDNA fragment. Since the probability of introducing a lesion in a small fragment is low, amplification of the 235 bp mtDNA fragment provides an accurate determination of the steady state of mtDNA levels. Then, the frequency of the mtDNA lesions was calculated using the Poisson equation.

The marked reduction in the POLG expression slows mtDNA replication and possibly affects mitochondrial biogenesis. The results reported here are in accordance with this assumption. In fact, the mtDNA copy number was significantly reduced in rats fed a fructose-rich diet (Figure 5), suggesting reduced mitochondrial biogenesis. Our finding of reduced PGC1α expression, the master regulator of mitochondrial biogenesis in rats fed a fructose-rich diet, also confirms this result (Figure 6). Further support to the reduced mitochondrial biogenesis in the liver of fructose-fed rats comes from the reduced expression of TFAM and NRF-1 found in these rats (Figure 6). In fact, TFAM is a mitochondrial transcription factor that is required to regulate the mitochondrial genome copy number [36], and NRF1 is supposed to control the key components of the protein import and assembly machinery [31], thus suggesting a broad meaning for NRF1 in orchestrating events in mitochondrial biogenesis. It is clear that a fructose-rich diet is associated with reduced mitochondrial biogenesis. The damage to hepatic mitochondrial integrity and mtDNA repair and replication mechanisms due to the fructose-rich diet could therefore lead to liver dysfunction, and consequently to metabolic diseases. Interestingly, it has been recently shown that exposure of skeletal muscle cells to fructose elicited damage similar to that found here by us in the liver, namely it increased oxidative stress and mitochondrial dysfunction, as well as decreased mitochondrial DNA content [37]. In contrast, Yamazaki et al. [38] found increased mitochondrial gene expression and mtDNA content in the liver of rats fed with fructose-containing drinking water. This different phenotype of mtDNA may be related to the severity of the metabolic syndrome and the components of the experimental design, such as the age of the rats at the start of the experiment and the duration of the treatment.

Indeed, several studies have shown that fructose consumption is associated with adverse alterations in the plasma lipid profile and severe hepatic steatosis, which is associated with necroinflammatory changes in mice [39,40,41]. Other studies have shown that rats fed a fructose-rich diet have increased hepatic triglyceride and cholesterol levels [42], and fructose fed to rodents at supraphysiological doses induced steatosis and steatohepatitis by de novo lipogenesis [43]. Meta-analyses have also suggested that fructose consumption is related to the risk factors for metabolic syndrome, such as increased triglyceride levels, stimulated hepatic de novo lipogenesis, and increased visceral fat [44,45,46,47].

Our current study showed that a fructose-rich diet leads to marked mtDNA damage; this diet also damaged the repair mechanism, resulting in a reduction of the mtDNA copy number and mitochondrial biogenesis. In addition, it can be speculated that mtDNA damage could lead to impairment in those systems that monitor mitochondrial health conditions and evoke appropriate stress responses, such as mitophagy, degradation of unfolded mitochondrial proteins and mitochondrial proteolysis [48].

In conclusion, our data indicate that long-term high fructose intake exerts deleterious effects on mitochondria, which may be an important factor contributing to the development of metabolic disorders, such as insulin resistance and steatohepatitis [49]. In addition, the present results could help to shed light on the recently evidenced cytotoxic effect of fructose and its metabolites in the induction of hepatocyte carcinogenesis [50].

## Figures and Tables

**Table 1:** Composition of experimental diets.

Component (g 100 g^−1^)	Control Diet	Fructose Diet
Standard chow *	50.5	50.5
Sunflower oil	1.5	1.5
Casein	9.2	9.2
Alphacel	9.8	9.8
Starch	20.4	-
Fructose	-	20.4
Water	6.4	6.4
AIN-76 mineral mix	1.6	1.6
AIN-76 vitamin mix	0.4	0.4
Choline	0.1	0.1
Methionine	0.1	0.1
Gross energy density, KJ·g^−1^	17.2	17.2
Protein, % metabolisable energy	29.0	29.0
Lipids, % metabolisable energy	10.6	10.6
Carbohydrates, % metabolisable energy	60.4	60.4
Of which: Fructose	-	30.0
Starch	52.8	22.8
Sugars	7.6	7.6

* Mucedola 4RF21; Italy.

**Table 2:** Body weight, plasma, and hepatic parameters in rats fed a control or a fructose-rich diet.

Item	Control	Fructose
Initial body weight, g	470 ± 10	470 ± 10
Final body weight, g	540 ± 23	545 ± 15
Food intake, g·day^−1^	32 ± 1.0	32 ± 1.0
Plasma ALT, U·L^−1^	16.8 ± 1.0	27.3 ± 1.0 *
Plasma AST, U·L^−1^	43.0 ± 3.1	65.2 ± 3.3 *
Hepatic lipid peroxidation, nmol TBARS·g^−1^ liver	61.5 ± 2.1	75.9 ± 2.0 *
Hepatic MPO activity, U·mg^−1^ liver	0.31 ± 0.01	0.62 ± 0.02 *

Values are the means ± SEM of nine different rats. * *p* < 0.05 compared to control diet. ALT = alanine transaminase, AST = aspartate transaminase, TBARS = thiobarbituric acid reactive substances, MPO = mieloperoxidase.
